# Western Diet-Induced Metabolic Alterations Affect Circulating Markers of Liver Function before the Development of Steatosis

**DOI:** 10.3390/nu11071602

**Published:** 2019-07-15

**Authors:** Daniela Gabbia, Marco Roverso, Maria Guido, Diana Sacchi, Michela Scaffidi, Maria Carrara, Genny Orso, Francesco Paolo Russo, Annarosa Floreani, Sara Bogialli, Sara De Martin

**Affiliations:** 1 Department of Pharmaceutical and Pharmacological Sciences, University of Padova, L.go Meneghetti 2, 35131 Padova, Italy; 2 Department of Chemical Sciences, University of Padova, via Marzolo 1, 35131 Padova, Italy; 3 Department of Medicine, General Pathology and Cytopathology Unit, University of Padova, via Giustiniani 2, 35128 Padova, Italy; 4 Department of Surgery, Oncology and Gastroenterology, University of Padova, via Giustiniani 2, 35128 Padova, Italy

**Keywords:** western diet, drug metabolism, lipid metabolism, NAFLD

## Abstract

Since nutrition might have a significant impact on liver function, we analyzed the early effect of Western-type diet on hepatic tissue and lipid and drug metabolism in Wistar–Kyoto rats (*n* = 8); eight rats fed with a standard diet were used as controls. Histological analysis of liver tissue was performed, and plasma biochemical parameters were measured. Plasma concentration of six bile acids was determined by ultra-liquid chromatography-tandem mass spectrometry UHPLC-MS/MS. Hepatic gene expressions of enzymes involved in drug and lipid metabolism were assessed by means of real-time reverse transcription (qRT)-PCR. Liver of rats fed with a Western diet did not show macroscopic histological alterations, but number and diameter of lipid droplets increased, as well as DGAT1, GPAT4, SCD, FASN and SREBP2 expression. Furthermore, Western diet-fed animals showed an increase in the activation of hepatic stellate cells and macrophage number in liver tissue, as well as a significant increase in AST and bilirubin levels (*p* < 0.01), and in the LDL:HDL cholesterol ratio (*p* < 0.001). Plasma chenodeoxycholic acid concentration increased significantly, whereas cholic acid decreased (*p* < 0.05), and cytochrome P450 genes were generally downregulated. Significant changes in hepatic lipid and drug metabolism are early induced by the Western diet, prior to steatosis development. Such changes are associated with a peculiar alteration in circulating bile acids, which could represent an early marker of non-alcoholic fatty liver disease (NAFLD) development.

## 1. Introduction

Food and beverages containing a high amount of fat and sugar are widely and commonly consumed [1]. The lack of physical activity [1], perinatal environment [1,2], and these diets are recognized as obesogenic [1], together with the presence of a predisposing genetic background [3]. In particular, consuming large amounts of added sugar, as, for instance, in soft drinks and colas, became extremely common in the 50s of the last century [4]. Fructose is frequently used as a sweetener in the preparation of different food and beverages [5], and it has been recently demonstrated [6] that consuming many fats and refined carbohydrates leads to augmented risk of developing different diseases, i.e., dyslipidaemia [7], obesity [5,8], insulin resistance [9], and heart disease [10]. In the liver, lipid accumulation may be due to the increased delivery of fatty acids or de novo lipogenesis, and/or decreased lipid clearance due to a drop of their secretion or oxidation [11,12,13,14,15]. The accumulation of fatty acids in the liver results in the development of non-alcoholic fatty liver disease (NAFLD), which currently represents one of the most common causes of chronic liver disease worldwide and one of the major causes of liver-related morbidity and mortality, and is strongly associated to the development of obesity, type 2 diabetes and metabolic syndrome [16,17]. Although the relative contribution of the different pathways described above to the development of NAFLD is only partially known, a number of clinical trials have demonstrated that the de novo lipogenesis plays a pivotal role in the development of NAFLD [18]. However, more insights are needed to understand the mechanism(s) underlying the transition between healthy and fatty liver.

The development of NAFLD is associated with alterations in the pool of circulating bile acids (BAs) both in patients and animal models [19]. BAs, steroid molecules synthesized in the liver starting from cholesterol, are actively secreted by hepatocytes into bile. The two primary BAs cholic acid (CA) and chenodeoxycholic acid (CDCA) are metabolized into secondary and conjugated BAs. Recently, BAs, their derivatives and compounds able to influence their signaling pathways have been found to be emerging candidate drugs for NAFLD and its complication non-alcoholic steatohepatitis (NASH) [19], since they may act as signaling molecules by interacting with a huge number of nuclear receptors (NRs), regulating their own synthesis and other metabolic processes, i.e., glucose, lipid, and drug metabolism.

On the basis of these considerations, the aims of this study were to evaluate whether the administration of a western diet (high-fat diet boosted with 30% fructose in drinking water) to Wistar Kyoto rats for a short term (12 weeks) affects (1) liver histology and function; (2) bile acid composition; (3) hepatic expression of the main drug-metabolizing enzymes and (4) hepatic lipid synthesis and metabolism.

## 2. Materials and Methods

### 2.1. Animal Model and Treatment

The procedures involving animals were conducted according to national and international regulations (Directive 2010/63/EU), and appropriate measures were used to minimize their pain or discomfort. The Ethics Committee of the University of Padova (OPBA) and the Italian Ministry of Health reviewed and approved all the protocols (Prot. no. 721, 2017), which fulfill completely the ARRIVE (Animal Research: Reporting of In Vivo Experiments) guidelines [20].

In this study, 16 male Wistar Kyoto rats (Charles River, Boston, MA, USA) were randomly assigned to two experimental groups: One group (standard diet rats, controls) consisted of animals (*n* = 8) fed with the standard diet used in the animal facility, whereas the others (Western diet rats, *n* = 8) were fed with a diet rich in fat (60/Fat, kcal from: 23.5% protein, 18.4% carbohydrate; 60.3% fat; Harlan Laboratories, Lesmo (Monza Brianza) Italy), boosted with 30% fructose in drinking water, as reported elsewhere [21,22]. Their body weight was measured once a week. After 12 weeks, the rats were sacrificed, and blood samples and livers were collected.

### 2.2. Reagents

Dimethyl sulfoxide (DMSO), 40% acrylamide solution, sodium chloride, sodium dodecyl sulfate (SDS), skimmed milk powder and Tween 20 were purchased from Sigma-Aldrich, Italy (Milan, Italy). Sucrose, Tris and magnesium chloride were obtained from Applichem (Chicago, IL, USA) and Complete Protease Inhibitor Cocktail from Roche (Milan, Italy). Ultrapure-grade water was produced with a Pure-Lab Option Q apparatus (Elga Lab Water, High Wycombe, UK). Mouse anti-CYP1A2, mouse anti-CYP3A1, rabbit anti-CYP3A2, mouse anti-CD-68 and HRP-conjugated anti-mouse antibodies were purchased from Abcam (Cambridge, UK). Mouse anti-CYP2B1/2B2 and mouse anti-βactin antibodies were obtained from Santa Cruz Biotechnology (Santa Cruz, CA, USA). Mouse anti- anti-α-smooth muscle actin (ASMA) antibody was purchased by Cell Marque (Rocklin, CA, USA). Cholic acid (CA), deoxycholic acid (DCA), chenodeoxycholic acid (CDCA), glycocholic acid (GCA), glycochenodeoxycholic acid (GCDCA), taurocholic acid (TCA), taurodeoxycholic acid (TDCA), taurochenodeoxycholic acid (TCDCA) and cholic acid-2,2,3,4,4-d5 (CA-d5—used as an internal standard—IS) were purchased from Sigma-Aldrich (St. Louis, MO, USA). All standard stock solutions were prepared by dissolving each bile acid in the appropriate amount of methanol to obtain individual stock solutions at 1 mg/mL. All solvents used for the chemical analysis were high performance liquid chromatography (HPLC) or high-performance liquid chromatography (LC–MS) grade from Sigma Aldrich (Milan, Italy). MilliQ water was obtained with a Millipore system (18.2 MΩ*cm).

### 2.3. Assessment of Liver Function and Immunohistochemical Study

In order to assess liver function, serum concentrations of albumin, ALT, AST, alkaline phosphatase (ALKP) and total and conjugated bilirubin were measured. Plasma lipid profile was assessed by measuring triglycerides, HDL-, LDL- and total cholesterol, and by calculating the LDL/HDL ratio.

Liver samples were processed using standard histological techniques and liver sections were stained with hematoxylin and eosin (H&E) [23] and examined by the same pathologist (MG), blinded to the study groups of origin of the liver sections.

### 2.4. Quantification of Gene Expression by qRT-PCR

Total RNA from liver tissue was obtained by means of a commercial RNA isolation kit (Promega Corporation, Madison, WI), as described in detail previously [24]. The gene expression of DGAT1, DGAT2, GPAT4, CYP1A2, CYP2B1, CYP3A1 and CYP3A2 was measured by real-time reverse transcription (qRT)-PCR using a one-step commercial kit (Takara, Mountain View, CA, USA), as reported elsewhere [20,24]. The relative mRNA expression was calculated according to the ∆∆Ct method [25]. β-actin was used as the housekeeping gene. The following primers (forward and reverse) were used in this study: CYP1A2 5′-tct gca gaa aac agt cca gga aca-3′, 5′-acc acc gtt gta cca ccg ttg t-3′; CYP3A1 5′-cca-tca-cgg-aca-cag-aaa-tg-3′, 5′-ctt-tcc-cca-taa-tcc-cca-ct-3′; CYP3A2 5′-agt-ggg-gat-tat-ggg-gaa-ag-3′, 5′-caa-tga-tgg-gga-aca-tct-cc-3′; CYP2B1 5′-aac gga ttc agg agg aag cc-3′, 5′-gcg ctc tcc aaa caca at gg-3′; DGAT1 5′-tcc tga att ggt gcg tgg tg-3′, 5′-gaa aca gag aca cca cct gga-3′; DGAT2 5′-gca gcg aga aca aga ata aag ga-3′, 5′-cca cct tgg atc tgt tga gc-3′; GPAT4 5′-tgt ggg acg gtg gat tga ag-3′, 5′-gct ccg gtc ctc atg gtt ac-3′ CYP7A1 5′-cgc acc tcg cta ttc tct gg-3′, 5′-tca gag gct gct ttc att gct-3′; CYP8B1 5′-ctg cac gta gcc agt acc aa-3′, 5′-gcc ttt ggg tcc tag cat ca-3′; CCL2 5′-tga tcc caa tga gtc ggc tg-3′, 5′-tgg acc cat tcc tta ttg ggg-3′; α-SMA 5′-cat cac caa ctg gga cga ca-3′, 5′-tcc gtt agc aag gtc gga tg-3′; COL1A1 5′-aaa acc acc aag acc tcc cg-3′, 5′-ggt ggg agg gaa cca gat tg-3′; SCD 5′-ggg tgc ctt atc gct ttc ct-3′, 5′-cag cct ctt gtc tac acc cg-3′; FASN 5′-gca ttt cca caa ccc caa cc-3′, 5′-aac gag ttg atg ccc acg at-3′; SREBP1 5′-cat gga cga gct acc ctt cg-3′, 5′-ggg cat caa ata ggc cag gg-3′; SREBP2 5′-cga act ggg cga tgg atg aga-3′, 5′-tct ccc act tga ttg ctg aca-3′; IL1β 5′-aaa tgc ctc gtc tgt ctg a-3′, 5′-caa ggc cac agg gat ttt gtc-3′; TNFα 5′-gat cgg tcc caa caa gga gg-3′, 5′-gct ggt ggt ttg cta cga c-3′ and β-ACTIN 5′-gcc-acc-agt-tcg-cca-tgg-a-3′, 5′-ttc-tga-ccc-ata-ccc-acc-at-3′.

### 2.5. Western Blot Analyses

Western blot analyses were run to measure the protein expression of four CYPs, i.e., CYP1A2, CYP2B1, CYP3A1 and CYP3A2 in the microsomal fraction using 30 µg of proteins per lane as described elsewhere [26]. Microsomal fractions of rat livers, whose protein content was assessed with a commercially available kit (Thermo Fisher BCA Protein Assay kit), were obtained as previously reported [27] and stored at −80 °C.

The following primary antibodies were used: Anti-CYP1A2 (1:2000), anti-CYP2B1 (1:2000), anti-CYP3A1 (1:4000), anti CYP3A2 (1:2000) and β-actin (1:2500). The signal intensity of the immunoreactive bands was normalized to that of beta-actin.

### 2.6. Immunofluorescence Coupled with Confocal Microscopy

Immunofluorescence was performed on Optimal Cutting Temperature (OCT) compound-embedded 5µm-sections, cut with a cryostat, followed by the detection of the antibody following the method described by De Martin et al. with some modifications [28]. Briefly, frozen sections were fixed with 4% PFA for 10 min and then permeabilized with 0.2% Triton X100 for 10 min at room temperature. Sections were washed in phosphate buffered saline (PBS), blocked with 5% FBS and incubated with the following primary antibodies: Mouse monoclonal anti-CD-68 (Abcam; dilution 1:500), as a marker of hepatic macrophages, and mouse monoclonal anti-α-smooth muscle actin (ASMA, Cell Marque; dilution 1:500), as a marker of hepatic stellate cells activation. Goat anti-mouse conjugated with fluorescent dye Alexa Fluor 568 was used as secondary antibody (Abcam). To stain cell nuclei, sections were incubated with 4′,6-diamidino-2-phenylindole (DAPI; 100 μg/mL, diluted 1:500) for 10 min. To detect lipid droplets (LDs), liver sections were incubated with the marker BODIPY 493/503 (Thermo Fisher; dilution 1:1000) for one hour at room temperature and mounted on glass slides with Mowiol 4–88 [29]. The images of the immunostained sections (five sections for each rat, for which at least three different fields were blindly analyzed) were acquired by a confocal microscope Zeiss LSM 800 (63× magnification) [30]. The intensity of the fluorescent signal was quantified by means of the ImageJ software (National Institutes of Health, Bethesda, MD, USA).

### 2.7. Bile Acid Extraction and LC-MS/MS Analysis

Bile acids were extracted from plasma samples as following described: 100 µL of plasma were treated with 400 µL of ice-cold acetonitrile spiked with 0.125 µM of IS, vortexed and centrifuged at 14800 rpm at 4 °C for 10 min. Supernatants were collected and injected into the LC-tandem mass spectrometric (LC-MS/MS) system, whose condition was already described in Gabbia et al. [20]. Each BA was quantified by using a five-point calibration curve, assayed in duplicate, prepared by spiking the matrix with the BAs of interest at final concentrations of 0.01, 0.05, 0.1, 0.5 and 1 µM, respectively, followed by the same extraction procedure used for the samples. We plotted the peak area ratios (A/A_IS_) related to the [M − H]^−^ extracted ion chromatogram of the selected BA and IS. Linearity showed an R^2^ > 0.99 for all the analytes. Limits of detection (LOD) of the method ranged from 0.0006 µM (0.31 µg/L) of TCA to 0.01 µM of CDCA, DCA (both 3.92 µg/L) and TCDCA (4.99 µg/L), with an overall reproducibility better than 12% as relative standard deviation (RSD).

### 2.8. Statistical Analyses

To calculate sample size (G*Power software 3.1, developed by the Heinrich-Heine-University, Düsseldorf, Germany), we assumed alpha = 0.05, power = 80%, and effect size = 1.4, and calculated that *n* = 8 animals per group were sufficient to evidence significant differences. Data were analyzed using the GraphPad Prism 7.0 software (GraphPad Software Inc., San Diego, CA, USA). The comparisons were performed by means of a student’ *t*-test for unpaired data. We considered statistically significant a *p*-value < 0.05.

## 3. Results

### 3.1. Effects of Western-Type Diet on Liver Histology, Body Weight and Plasma Biochemistry

**Figure 1:**
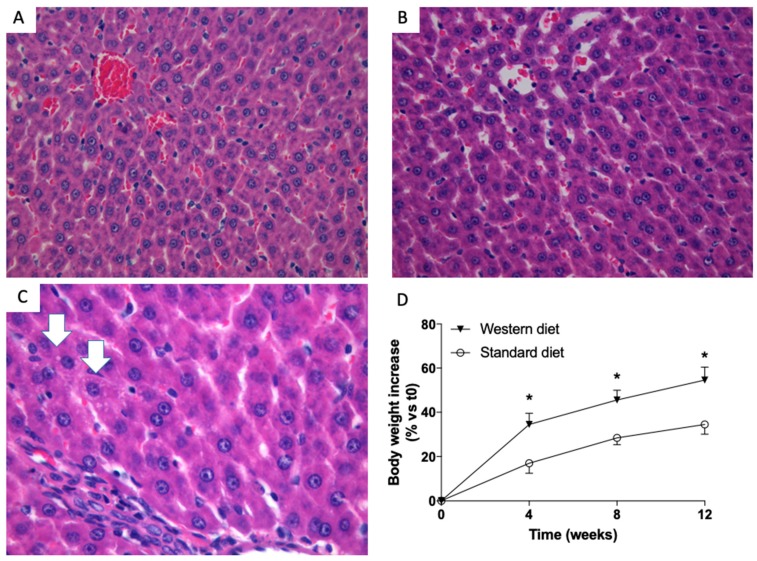
Liver histology of rats. Representative photomicrographs of liver tissue from a rat fed with a standard (**A**) and Western (**B**) diet (hematoxylin and eosin—H&E staining,). At 40× magnification, the histology of rats fed with a standard and Western diet were indistinguishable. At 63× magnification, small droplets were present in some hepatocytes of Western diet-fed rats (**C**, white arrows). Western-diet induced an increase in rat body weight. (**D**) Significantly higher body weight increases in rats fed with a western diet (triangles) diet vs. rats fed with standard diet (dots). * *p* < 0.05 vs. rats fed with standard diet.

When performed at low magnification, liver histology failed to show any significant change and livers from control (Figure 1A) and study (Figure 1B) group were indistinguishable. However, if analyzed at a very high magnification, some hepatocytes with very subtle droplets were detected in the liver sections of animal fed with Western diet (Figure 1C). The body weight of all the animals was measured once a week. Figure 1D shows that the body weight of rats fed with a Western Diet increased more than that of rats fed with the standard diet, and this increase started to be significant (*p* < 0.05) four weeks after the beginning of the treatment.

Biochemical parameters were indicative of an impairment of liver function: AST (*p* < 0.05) and both total and direct bilirubin (*p* < 0.01) increased significantly in rats fed with a Western Diet. Furthermore, the plasma lipid profile was also affected by a Western Diet administration, since triglycerides (*p* < 0.05) and LDL cholesterol (*p* < 0.001), as well as the LDL:HDL ratio (*p* < 0.01) were significantly higher in rats fed with this diet with respect to controls (Table 1).

### 3.2. Effect of a Western-Type Diet on Hepatic Lipid Droplets and Hepatic Liver Metabolism

**Figure 2:**
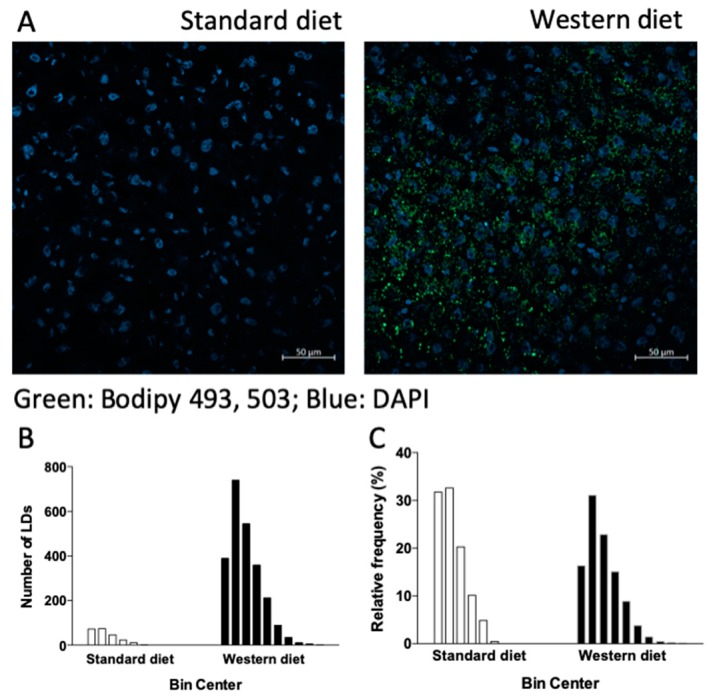
Lipid droplets (LDs) in rat livers. Staining with Bodipy 493/503, a marker of lipid droplets. Cell nuclei are stained blue with 4′,6-diamidino-2-phenylindole (DAPI). (**A**). Absolute number of the different classes of LDs according to their diameter (observed range: 0.4–2.4 μm and 0.4–4 μm for rats fed with standard and Western diet, respectively) (**B**), and their relative frequencies (**C**).

Since in liver cells neutral lipids are stored in lipid droplets (LDs), dynamic organelles whose pivotal role in the pathogenesis of liver steatosis has been extensively demonstrated (see [31] and refs therein), we incubated the liver sections of rats fed with a standard or Western diet with the LD marker BODIPY 493/503. In accordance with the histological findings, Figure 2 shows that the number of LDs was significantly higher in rats fed with a Western diet, as clearly shown by the increase in green fluorescence (Figure 2A) and in the absolute number of LD, as indicated by the frequency analysis reported in Figure 2B. The relative frequency of the different classes of LDs in terms of diameter (starting from 0.4 μm with increments of 0.4 μm for each class, Figure 2C) indicates that, in rats fed with a Western diet, hepatic LDs are also characterized by a higher dimension with respect to those of rats fed with a standard diet. The formation of extremely large LDs inside hepatocytes is the hallmark of steatosis. In order to gain more information on lipid synthesis, accumulation and metabolism in the liver, we measured the mRNA expression of different genes involved in these pathways.

**Figure 3:**
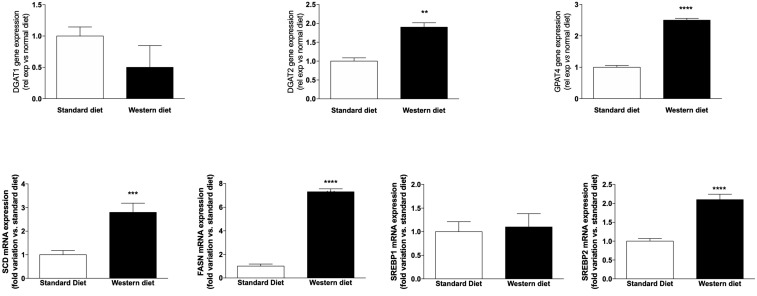
mRNA expression of genes involved in lipid synthesis and metabolism. ** *p* < 0.01, *** *p* < 0.001 and **** *p* < 0.0001 vs. rats fed with standard diet.

According to histological and immunofluorescence data, Figure 3 shows that the mRNA expression of two genes, which are responsible for the synthesis of the triglycerides stored in LDs, i.e., diglyceride acyltransferase 2 (DGAT2) and glycerol-3-phosphate acyltransferase (GPAT4), increased significantly (*p* < 0.01 and *p* < 0.0001, respectively) in animals fed with a Western diet. Furthermore, stearyl-CoA desaturase (SCD) and fatty acid synthase (FASN), two genes playing an important role in lipid biosynthesis, increased significantly in rats fed with a Western Diet (*p* < 0.001 and *p* < 0.0001, respectively). The mRNA expression of the isoforms 1 and 2 of sterol regulatory-element binding protein (SREBP) was either unchanged (SREBP1) or increased significantly (SREBP2, *p* < 0.0001).

### 3.3. Effect of Western-Type Diet on the Activation of Hepatic Stellate Cells and Macrophages

**Figure 4:**
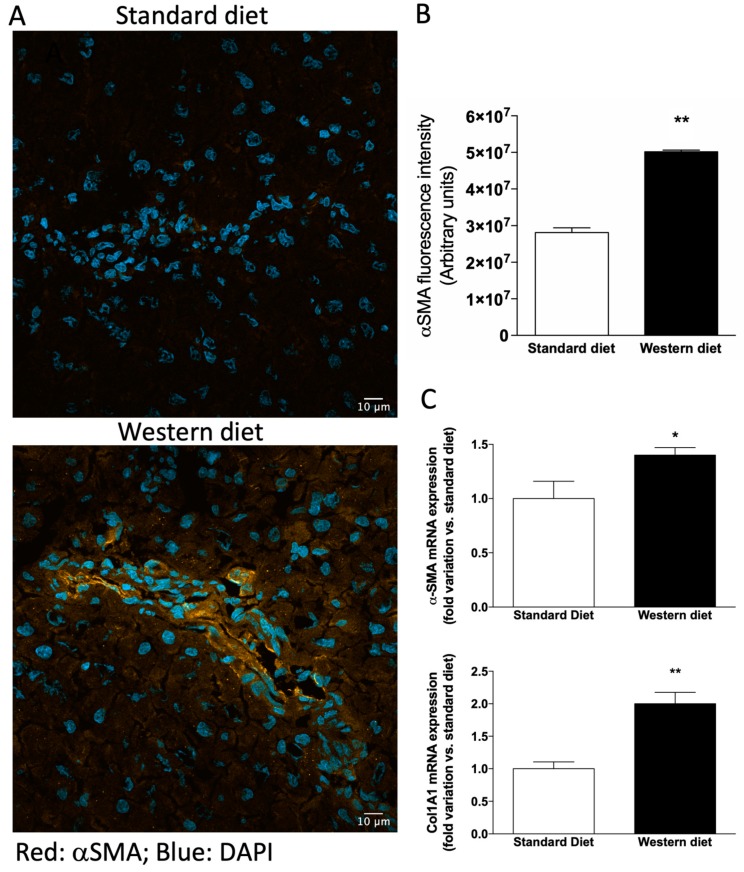
Activation of hepatic stellate cells. (**A**) Representative photomicrographs of liver tissue obtained from a rat fed with a standard (upper panel) and Western (lower panel) diet stained with α−SMA, a marker of hepatic stellate cells (HSCs) activation. (**B**) Quantification of α−SMA-related immunofluorescence. Values are reported as means ± S.E.M. Representative images from five independent experiments are shown. Cell nuclei are stained blue with DAPI. (**C**) mRNA expression of the fibrosis markers α−SMA and Col1A1. * *p* < 0.05 and ** *p* < 0.01 vs. rats fed with standard diet.

Since we observed alterations in biochemical markers of liver function in rats fed with a Western diet, although liver histology was normal, we performed an immunofluorescence analysis to ascertain whether hepatic stellate cells (HSCs) were activated in these rats, because activated HSCs represent the primary source of myofibroblasts leading to liver fibrosis in several animal models of hepatotoxic liver injury [32]. As shown in Figure 4A,B, the α-SMA-related fluorescence increased significantly in rats fed with a Western diet with respect to controls (*p* < 0.01). Accordingly, the mRNA levels of both α-SMA and the other fibrosis marker Col1A1 increased significantly in Western diet rats (Figure 4C, *p* < 0.05 and *p* < 0.01, respectively).

**Figure 5:**
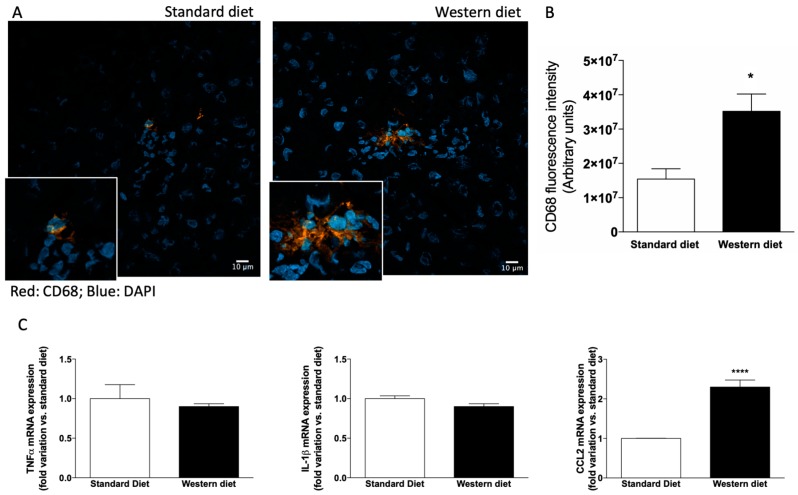
CD-68 positive cells in the liver. (**A**) Representative photomicrographs of liver tissue obtained from a rat fed with standard (upper panel) and Western (lower panel) diet stained with CD-68, a marker of hepatic macrophages. (**B**) Quantification of CD-68-related immunofluorescence. Values are means ± S.E.M. The histograms report the intensity of fluorescence (arbitrary units). Representative images from five independent experiments are shown. Cell nuclei are stained blue with DAPI. (**C**) mRNA expression of genes involved in the inflammatory process. * *p* < 0.05, **** *p* < 0.0001 vs. rats fed with standard diet.

Since HSCs are activated upon stimulation with cytokines secreted from resident and infiltrating cells [33], we also evaluated the presence of CD68-positive cells in the liver. CD68 is an indicator but not an exclusive marker of Kupffer cells, because also monocyte-derived macrophages infiltrating the liver may express this antigen, and preclinical and clinical studies provide convincing evidence for the pivotal role of macrophages in the development and progression of NAFLD [34] as shown in Figure 5A,B, CD-68 related fluorescence increased in the liver of rats fed with a Western diet (*p* < 0.05), indicating either an activation of Kupffer cells or an infiltration of the hepatic tissue by monocyte-derived macrophage, or both. Accordingly, a significant increase of the mRNA expression of the inflammatory chemokine CCL could be observed in the liver of rats fed with a Western diet (Figure 5C).

### 3.4. Effect of a Western-Type Diet on the Circulating Pool of Bile Acids

**Figure 6:**
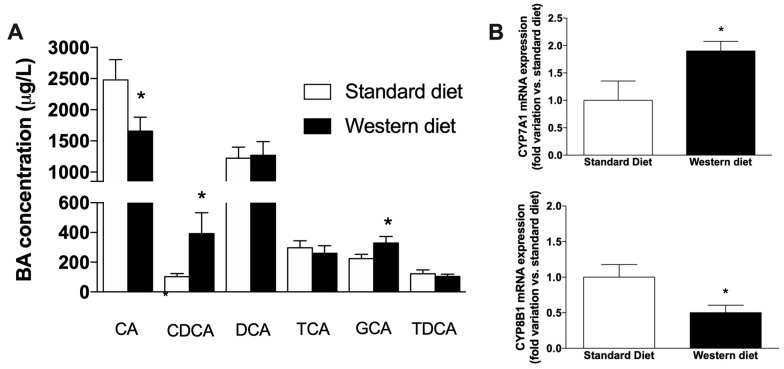
Analysis of plasma bile acids. (**A**) Alteration of circulating BAs induced by the administration of a Western diet. (**B**) Hepatic mRNA expression of genes involved in BA synthesis. * *p* < 0.05 vs. rats fed with standard diet.

We performed a chemical analysis of plasma bile acids (BAs), with the aim of ascertaining whether peculiar alterations of BAs circulating pool could be observed after Western Diet administration, even in the absence of liver steatosis. As shown in Figure 6A, the administration of Western Diet significantly affects the plasma concentration of the two primary BAs, i.e., CA, which decreases significantly in Western Diet rats (*p* < 0.05 vs. controls) and CDCA, which, on the contrary, significantly increases (*p* < 0.05 vs. controls). Of the other secondary and conjugated BAs measured, only the concentration of GCA was significantly affected by Western Diet consumption (*p* < 0.05 vs. controls). We also measured the mRNA expression of two main genes involved in the synthesis of BAs (CYP7A1 and CYP8B1, Figure 6B), finding an opposite effect of the Western diet on their expression, i.e., CYP7A1 significantly increased (*p* < 0.05 vs. controls), whereas a drop of CYP8B1 expression could be observed with respect to rats fed with a standard diet (*p* < 0.05).

### 3.5. Effect of a Western-Type Diet on Hepatic Drug Metabolism

**Figure 7:**
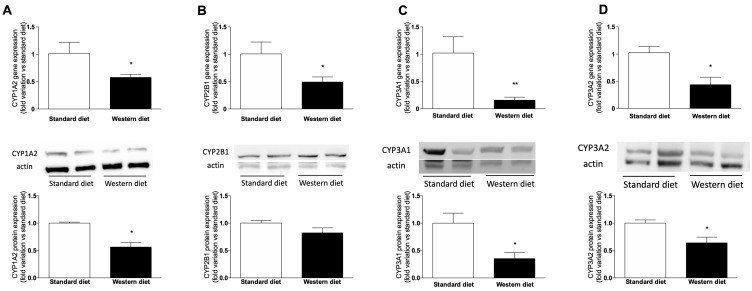
mRNA and protein expression of CYPs. mRNA (upper panel) and protein (lower panel) expression of CYP1A2 (**A**), CYP2B1 (**B**), CYP3A1 (**C**) and CYP3A2 (**D**). Between graphs, representative western blot analyses have been reported. The analysis was performed on cell lysates obtained from eight animals per group. * *p* < 0.05, ** *p* < 0.01 vs. rats fed with standard diet.

Since we observed a change in the expression of the two isoform of cytochrome P450 (CYP) involved in BA synthesis, i.e., CYP7A1 and CYP8B1, and it is well-known that BAs can regulate drug-metabolizing enzymes by activating the nuclear receptors responsible for their transcription, we ascertained whether the short-term administration of a Western Diet affects hepatic drug metabolism, by measuring gene and protein expressions of the main CYP isoforms responsible for the metabolism of drugs, i.e., CYP1A2, CYP2B1, CYP3A1 and CYP3A2 (the latter are the orthologues of human CYP3A4, [35]). Figure 7 shows that the expression of all the analyzed genes decreased significantly in rats fed with a Western Diet. Similar results have been obtained for the protein expression of CYPs, since CYP2B1 protein expression tended to decrease in animals fed with a Western diet with respect to standard-diet-fed rats, without reaching the statistical significance (Figure 7B), whereas the other three proteins (CYP1A2, Figure 7A, CYP3A1, Figure 7C and CYP3A2, Figure 7D) were significantly downregulated (*p* < 0.05) in rats fed with a Western Diet.

## 4. Discussion

In this study, we analyzed the effect of the short-term administration of a diet rich in fat and refined sugar (Western diet) on the liver of Wistar Kyoto rats, a strain which is prone to develop liver steatosis when treated with diets rich in fat [36,37,38]. Although a Western diet, when consumed by humans, is typically characterized by high amounts of saturated fats, proteins from red meats, refined sugars such as sucrose and fructose and sodium, in animal studies the term Western diet is used for indicating a rodent diet that is higher in fat or manipulated with the aim of inducing chronic diseases associated with the Western human dietary pattern [39]. In this work, we used a diet rich in fat and fructose to obtain a model of pre-steatosis, since the administration of this kind of diet for periods longer than 12 weeks to mice or rats (see e.g., [40] and refs therein) leads to the development of NAFLD, a liver disease associated with a Western diet and lifestyle. We decided to perform a short-term treatment in order to catch early alterations of the hepatic tissue that can probably lead to liver steatosis and identify putative markers of the transition from a healthy to a fatty liver. Our results clearly demonstrate that liver function tests, lipid metabolism as well as drug metabolism, are subjected to significant changes by Western diet consumption before evident histological liver lesions develop.

Even in the absence of macrovescicular steatosis, animals fed with a Western Diet had a significantly higher AST and bilirubin levels. The relationship between plasma bilirubin and NAFLD is controversial. Indeed, although mildly elevated serum bilirubin levels were reported to reduce the risk of developing this disease [41,42], a recent study [43] provided evidences against this hypothesis. Therefore, the observed increase in total and direct bilirubin, together with the one of AST, is probably related to an early impairment of liver function.

The observed changes in plasma lipid profile are not surprising, because it is well-known that consuming a diet rich in fat and sugar leads to an increase in triglycerides and LDL cholesterol, and to an increase of body weight. In turn, changes in circulating cholesterol can cause alterations in the pool of bile acids, since the first precursor of their synthetic pathway is cholesterol itself. An increased concentration of the primary bile BAs CA and CDCA [44], with a consequent increase of their ratio, has been evaluated as a marker of intrahepatic cholestasis of pregnancy [45] and progressive familial intrahepatic cholestasis [46]. More recently, changes have been observed in animal models and patients affected by NAFLD and NASH [47,48]. Coherently, we also observed a significant increase of the CDCA drop of CA plasma concentration in rats fed with a Western diet, but there was a significant drop of CA, leading to a decrease of the CA:CDCA ratio. A possible explanation of this peculiar effect is the reduced expression of CYP8B1 we observed in the liver of rats fed with a Western diet. In fact, this isoform of cytochrome P450 is known to be the crucial regulator of the balance of CA and CDCA in the liver by converting CDCA to CA [49]. In contrast, CYP7A1 mRNA expression increased in Western diet rats, indicating that the synthesis of bile acids is generally increased in these rats, as also observed in obese subjects and in NAFLD/NASH patients [50], further suggesting that the reduction of CA is due to CYP8B1 downregulation, since CYP7A1 catalyzes the first and common step of primary BA synthesis. Furthermore, we found that four CYP isoforms responsible for drug metabolism (CYP1A2, CYP2B1, CYP3A1 and CYP3A2) were transcriptionally downregulated in rats fed with a Western diet. Numerous studies have shown a significant effect of NAFLD on expression and activity of drug metabolizing enzymes in animal models [51,52]. Accordingly, in vitro studies in primary human and animal hepatocytes obtained from livers with or without steatosis showed that this condition has a profound impact on the metabolic functionality of hepatocytes [53,54]. It has been observed that deposition of fat in human hepatocytes can lead to a marked reduction in CYP mRNA and activity [55]. Furthermore, considerable changes in hepatic uptake, distribution, metabolism and transport of drugs have been observed in NAFLD/NASH patients, due to changes in biliary excretion, systemic concentrations and renal handling of different drugs, and these modifications lead to alterations in drug efficacy and/or toxicity ([56] and refs therein).

It should be noticed that in this study we used only male rats. However, several studies reported a significant effect of gender on CYP expression in different animal models, i.e., rodents [57], and, more recently, zebrafish [58]. Further studies are needed to ascertain whether a Western diet effect on drug-metabolizing enzymes is related to gender and to analyze the translational impact of this finding. Interestingly, we observed that in male rats the reduction of drug-metabolizing enzymes is present even before the development of steatosis, and it is strictly related to the consumption of a diet rich in fat and refined sugar. Notably, although glucose and fructose are equally added to beverages, it is getting clear that the latter is potentially more harmful, since, unlike glucose, fructose is preferentially subjected to hepatic metabolism [59]. This and other features of its metabolism make it extremely lipogenic [60,61]. Based on this consideration, we analyzed how hepatic lipid metabolism was affected by Western diet administration before the development of steatosis. Initially, since histological analysis revealed small droplets in some hepatocytes of rats fed with a Western diet, we focused on determining the number and size of LDs in the liver and demonstrate that the administration of Western diet increases significantly number and size of LDs, although we did not measure hepatic triglycerides content by biochemical methods. Accordingly, two of the enzymes responsible for triacylglycerol synthesis, i.e., DGAT2 (but not DGAT1) and GPAT4 increased significantly in rats fed with a Western diet. It is well known that DGAT1 and 2 have distinct roles in lipid metabolism. In particular, recent observations demonstrated that DGAT2 catalyzes the de novo synthesis of triacylglycerols, giving to this enzyme a pivotal role in the development of carbohydrate-induced hypertriglyceridaemia and, consequently, hepatic steatosis. DGAT1 has the complementary ability of rescuing partial glycerides from hydrolysis [62]. As far as GPAT4 (a microsomal GPAT isoform) is concerned, it has been demonstrated that its overexpression in hepatocytes causes an impaired glucose homeostasis [63]. Furthermore, we observed that the mRNA expression of SCD and FASN, two enzymes involved in the synthetic pathway of fatty acids, was increased, suggesting a rise of also fatty acid synthesis in rats fed with a Western diet. This can be probably postulated also for cholesterol synthesis, since we measured an increase of the mRNA expression of SREBP2, a protein known to modulate cholesterol biosynthesis, besides regulating fatty acid synthesis, as recently described [64]. Taken together, our results on lipid synthesis and metabolism alterations indicates that lipid accumulation and de novo lipogenesis are increased early in rats fed with a Western diet, prior to the development of hepatic steatosis. Interestingly, although the histology evidenced the absence of macroscopic alterations, the increases of α-SMA- and CD68-positive cells in the liver, although limited, indicate that a mild activation of HSCs (also confirmed by the increase of α-SMA and Col1A1 mRNA expression in liver tissue, and consistent to what has been observed analyzing the transcriptome of obese and NAFLD/NASH patients [50]), together with a slight increase in liver macrophages are present in the liver of rats fed with a Western diet, and the increased number of LDs could be responsible of this effect. Accordingly, we demonstrated an increase of the mRNA expression of CCL2, a chemokine with a target action to macrophages playing a pivotal role in the liver during early stages of inflammation and fibrosis, especially when these inflammatory processes are linked to NAFLD or NASH [65].

## 5. Conclusions

In conclusion, our data indicate that, in the liver, lipid metabolism as well as drug metabolism are subjected to significant changes by Western diet consumption before the development of steatosis. This kind of diet can therefore be considered as a possible source of relevant drug metabolic interactions. Furthermore, the pool of circulating bile acids also shows a significant alteration, which can be evaluated as a putative early marker of NAFLD development.

## Figures and Tables

**Table 1:** Biochemical parameters of liver function in standard and Western diet rats.

	Standard Diet	Western Diet
Albumin (g/L)	41.05 ± 2.44	43.53 ± 2.26
ALKP (U/L)	167.5 ± 37.3	144.0 ± 11.1
ALT (U/L)	58.33 ± 11.25	63.67 ± 8.39
AST (U/L)	163.6 ± 36.4	265.0 ± 61.9 *
Total bilirubin (mmol/L)	0.92 ± 0.32	1.88 ± 0.32 **
Direct bilirubin (mmol/L)	0.67 ± 0.31	1.37 ± 0.12 **
Triglycerides (mmol/L)	0.57 ± 0.19	0.97 ± 0.11 *
Total Cholesterol (mmol/L)	2.18 ± 0.19	2.38 ± 0.06
HDL Cholesterol (mmol/L)	1.59 ± 0.20	1.73 ± 0.09
LDL Cholesterol (mmol/L)	0.35 ± 0.03	0.50 ± 0.05 ***
LDL:HDL ratio	0.22 ± 0.02	0.29 ± 0.03 **

Results are given as mean ± S.D. Alkaline phosphatase (ALKP), Alanine transaminase (ALT), aspartate transaminase (AST), high-density lipoprotein (HDL) and low-density lipoprotein (LDL). * *p* < 0.05, ** *p* < 0.01 and *** *p* < 0.001 vs. rats fed with standard diet.
